# The impact and predictive value of refeeding syndrome on the short-term prognosis of patients with severe stroke: a retrospective cohort study

**DOI:** 10.3389/fnut.2026.1682717

**Published:** 2026-02-12

**Authors:** Wei Zhang, Shengxiang Zhang, Yun Tang, Tao Yu

**Affiliations:** 1 Department of Rehabilitation, The First Affiliated Hospital of Wannan Medical College, Wuhu, Anhui, China; 2 Department of Neurosurgery, Intensive Care Unit, The First Affiliated Hospital of Wannan Medical College, Wuhu, Anhui, China

**Keywords:** hypophosphatemia, nutrition, prognosis, refeeding syndrome, stroke

## Abstract

**Introduction:**

Refeeding syndrome (RFS) may have adverse effects on patients. However, the impact of RFS on the prognosis of severe stroke patients remain unclear. This study aims to explore the impact on short-term prognosis of patients with severe stroke.

**Methods:**

A total of 305 patients were included for retrospective analysis. The electrolyte changes and short-term prognosis between RFS and Non-RFS (NRFS) groups of patients were compared. Besides, the risk factors for prognosis of patients and the predictive efficacy of RFS in patient prognosis were analyzed.

**Results:**

The new acute liver injury (ALI), new acute kidney injury (AKI), new mechanical ventilation (MV), mortality within 28 d, and mRS scores at the discharge were higher in the RFS group than in the NRFS group (*p* < 0.05). The serum phosphorus level in the RFS group decreased after EN compared to before EN (*p* < 0.05). The area under the ROC curve (AUC) of RFS for predicting poor prognosis in patients with severe stroke was 0.678, with a sensitivity of 64.7%, and a specificity of 61.4%.

**Conclusion:**

RFS is an independent risk factor for the patient’s functional prognosis, therefore, medical staff should screen high-risk populations as early as possible and provide preventive treatment according to the guidelines.

## 1 Introduction

Stroke is a major global health issue characterized by high mortality and disability. This study explores the impact of Refeeding Syndrome (RFS) on short-term outcomes in patients with severe stroke.

Stroke patients have a high incidence of malnutrition due to functional impairments such as consciousness, cognition, swallowing caused by impaired neurological function, as well as neuroendocrine dysfunction and gastrointestinal stress disorder (1). In addition, patients with severe stroke are in a state of stress due to severe cerebrovascular disease, surgery, and infection. This leads to increased release of inflammatory cytokines in the body, abnormal hormone metabolism, and accelerated fat and glycogen breakdown. At the same time, a large amount of protein in the body is broken down autonomously, and oxidative stress will inhibit protein synthesis, which is more likely to lead to malnutrition (2). Shoeibi et al. (3) reported that the incidence of malnutrition in patients with acute severe stroke can reach as high as 34%. Malnutrition can easily lead to impaired immune function and multi system metabolic disorders in the body, which have a negative impact on the recovery of patients’ neurological function, resulting in prolonged hospitalization and increased mortality (4). Therefore, nutritional support plays an important role in the recovery of patients’ conditions and is a prerequisite for the treatment of patients with severe stroke.

At present, the main nutritional support treatment for severe stroke patients is enteral nutrition (EN), which can not only improve the nutritional status, but also can maintains the stability of the internal environment and reduces in-hospital mortality in neurocritically ill patients (5, 6). However, a series of complications may occur during the process of nutritional support therapy, among which RFS has been discovered in recent years. American Society for Parenteral and Enteral Nutrition (ASPEN) defined RFS as a 10% reduction in serum levels of at least one electrolyte (potassium, magnesium, and/or phosphate) within 5 d of initiation of nutritional support and/or organ dysfunction caused by a reduction in these electrolytes and/or secondary to thiamine deficiency (7). The characteristic of RFS is hypophosphatemia, which may lead to cardiac, respiratory, hematologic, neurological and other organ systems dysfunction and even death (8). Mostly, RFS occurs in the first 3 days after nutritional support. All types of nutritional support have a risk of developing RFS, with EN or parenteral nutrition is the highest risk (9, 10). Therefore, due to a series of adverse effects of patients caused by RFS, the prevention and treatment of RFS is also important (11, 12).

However, due to the diverse clinical manifestations and lack of specificity of RFS, it is difficult to accurately define it, resulting in varying incidence rates reported in previous studies. Studies have shown that the incidence of RFS in critically ill patients is 34–74.2% (13, 14), and the incidence of RFS in neurocritical patients is 17.1–50.1% (15, 16). ASPEN suggested to pay attention to preventing RFS in patients with severe malnutrition7. Previous studies mainly focus on the screening of high-risk populations for RFS, exploring the risk factors of RFS and developing risk prediction models of RFS. Studies have shown that the age of critically ill patients, low serum albumin concentration (<30 g/L), low serum prealbumin concentration (<150 g/L), history of diabetes, nasointestinal feeding, high temperature of enteral nutrient solution, fast feeding speed, nutrition risk screening (NRS 2002) score ≥3, and sequential organ failure score (SOFA) are all risk factors for RFS in critically ill patients (16–18). In addition, some scholars have screened risk factors of RFS and incorporated them into risk prediction equations to develop risk prediction models of RFS, and all of which have good predictive performance (15, 19). These studies believed that risk prediction models of RFS for critically ill patients can effectively improve the accurate identification of RFS in different diseases by clinical medical staff, and provide reference for further prevention of RFS. However, it is worth noting that there are few studies on the prognostic effects of RFS on severe stroke patients. In view of this, this study retrospectively analyzed the correlation between RFS and short-term prognosis of severe stroke patients, in order to provide reference for avoiding adverse clinical outcomes caused by RFS on patients.

## 2 Materials and methods

### 2.1 Study design

This is a retrospective analysis of severe stroke patients admitted to the Neurosurgery ICU (NSCU) of a tertiary comprehensive hospital in China from January 2021 to May 2022.

### 2.2 Study population

We selected adult stoke patients admitted to the NSICU by using convenience sampling. The inclusion criteria of patients: (1) Hemorrhagic stroke or ischemic stroke diagnosed by physical examination and CT or MRI (20); (2) Age ≥ 18 years and<80 years old; (3) Patients with nutritional risk identified based on the Nutrition Risk Screening and Evaluation Form [NRS 2002 (21)] (NRS 2002 score ≥ 3 points); (4) The GCS score is ≤ 12 points or the NIHSS score is ≥ 11 points at admission; (5) Enteral nutrition treatment during admission to NSICU for ≥ 72 h. (6) Patients or their family informed consent. The exclusion criteria of patients: (1) Patients with renal insufficiency or any form of blood purification during treatment; (2) Patients with moderate to severe liver dysfunction (peripheral blood alanine aminotransferase/aspartate aminotransferase increased by more than three times the normal value) (22); (3) Patients with a history of parathyroidectomy or a history of hyperphosphatemia in the past 3 months; (4) Factors affecting the adverse prognosis of patients, including heart disease, cerebral hernia, coagulation disorders, or gastrointestinal dysfunction; (5) Incomplete medical record data.

### 2.3 Grouping criteria

The sample was divided into RFS group and NRFS group based on whether it meets the RFS diagnostic criteria. In this study, participants were neurocritical patients. Therefore, we used the diagnostic criteria for RFS by Doig et al. (23), which defined RFS in critically ill patients as newly developed hypophosphatemia within 72 h after nutritional support, with serum phosphorus <0.65 mmol/L and a decrease from baseline >0.16 mmol/L.

According to the evaluation results of the modified Rankin Scale (mRS) (24), patients were divided into a poor prognosis group (≥ 3 points or death) and a good prognosis group (<3 points).

### 2.4 Data collection

Two researchers reviewed the original medical records of patients admitted to NSICU (with 18-beds) during the study period. The double-blind principle applied during data collection, blood biochemical analysis, and scale scoring. Based on the inclusion and exclusion criteria in this study, as well as the RFS diagnostic criteria, patients were independently screened and cross checked. All data was collected using a self-designed questionnaire and retained by a dedicated staff. The demographic information of patients is obtained through electronic medical records. The GCS, APACHE II, and SOFA scores of patients at admission to the NSICU were evaluated and recorded. In addition, electrolyte test results were obtained within 24 h of admission to the NSICU and within 72 h after receiving EN, including serum potassium, serum sodium, serum phosphorus, and serum magnesium. At the same time, any clinical complications and prognostic indicators within 28 d after nutritional therapy were recorded, including ALI (alanine aminotransferase 3 times or more above normal value) (25), AKI (a rise in blood creatinine of 26.5 mmol/L within 2 d or 1.5 times the baseline value within 7 d) (26) that meet the diagnostic criteria, new MV, mortality rate within 28 days, length of hospitalization and mRS score on discharge.

### 2.5 Sample size

The sample size was calculated using the EPV method, and the recommended empirical guideline in logistic regression is an EPV of at least 10 to ensure robust results (27), and the calculation formula is: *n* = independent variable × 10/prevalence rate. A total of 11 independent variables were included in this study, and a literature search yielded a prevalence of RFS in neurocritical care patients ranging from 17.1–50.1% (13, 14). Therefore, the sample size for this study according to the calculation formula was 220–643 cases.

### 2.6 Ethical approval

This is a retrospective study and we did not perform additional interventions beyond the necessary assessments. Verbal consent was obtained from all patients or their families after being informed of the situation. All the data in this study were obtained from the electronic medical record system, and the data were obtained and used in accordance with the relevant regulations and ethical guidelines. The ethical review of this study was conducted by the Ethics Committee of The First Affiliated Hospital of Wannan Medical College.

### 2.7 Statistical analysis

Data were analyzed by using SPSS Statistics version 22.0 software (IBM, Armonk, New York, United States). Normally distributed, continuous variables were represented as mean ± standard deviation (SD), while results for non-normally distributed, continuous variables were presented as median [interquartile range (IQR)], Categorical variables are represented as counts and percentage. The baseline demographic characteristics, type of disease, comorbidities, baseline assessment, and the biochemical indicators compared between the good prognosis and poor prognosis groups; prognostic indicators compared between RFS and NRFS groups by using the Mann–Whitney U tests for continuous variables and Chi-Square test for categorical variables. Differences of changes in electrolyte levels before and after EN treatment were analyzed using matched samples t-test; Binary logistic regression was used to analyze the correlation between RFS and prognosis of patients. Due to the receiver operating characteristic (ROC) curves can assess the performance of the model effectively and provide an intuitive visualization tool. Therefore, the predictive efficacy of RFS on patient prognosis was validated through ROC area under the curve (AUC). *p* < 0.05 indicates a statistically significant difference. Cumulative survival was compared between the two groups using Kaplan–Meier survival analysis, and the multivariate cox regression was applied to screen for independent factors affecting survival in patients with severe stroke.

## 3 Results

### 3.1 Comparison of clinical data between the different prognoses groups

Comparison of age, type of disease, APACHE II, SOFA, GCS score, prealbumin on admission, and RFS between patients in the poor prognosis group and the good prognosis group, the difference was statistically significant (*p* < 0.05). The results are displayed in Table 1.

**Table 1:** Comparison of the demographics of patients between the different prognoses groups.

Categories	GP(*n* = 70)	PP (*n* = 235)	*t*/*χ*^2^ value	*p-*value
Male, *n* (%)	43(61.43)	143(60.85)	0.008	0.931
Age (^−^*x* ± *s*, years)	54.21 ± 13.14	61.22 ± 12.29	−4.118	0.000
Type of disease, *n* (%)				
Haemorrhagic stroke	62(88.57)	224(95.32)	4.204	0.040
Ischaemic stroke	8(11.43)	11(4.68)		
Comorbidity, *n* (%)				
Hypertension	29(41.43)	93(39.57)	0.077	0.781
Diabetes	5(7.14)	18(7.66)	0.021	0.886
Cerebrovascular diseases	3(4.29)	18(8.33)	0.958	0.328
Baseline assessment (^−^*x* ± *s*, score)				
APACHE II	13.99 ± 5.22	16.27 ± 4.75	−3.451	0.001
SOFA	5.74 ± 1.69	6.81 ± 2.54	−3.306	0.001
GCS	6.97 ± 2.58	7.92 ± 3.27	−2.236	0.026
Biochemical indicators (^−^*x* ± *s*)				
Serum albumin (g/L)	37.25 ± 6.00	36.02 ± 4.70	1.809	0.071
Prealbumin (g/L)	20.97 ± 6.13	19.30 ± 5.64	2.132	0.034
Creatinine (umol/L)	65.92 ± 43.31	68.07 ± 56.18	−0.295	0.768
RFS, *n* (%)	12(17.14)	77(32.77)	6.370	0.012

GP, good prognosis; PP, poor prognosis.

### 3.2 Comparison of the prognosis of patients between the RFS and the NRFS groups

The new ALI, new AKI, new MV, mortality within 28 d, and mRS scores at the discharge were higher in the RFS group than in the NRFS group, the difference was statistically significant (*p* < 0.05); while the difference in the length of hospitalization between the two groups was not statistically significant (*p* > 0.05), which as shown in Table 2.

**Table 2:** Comparison of the prognosis of patients between the case and control groups.

Categories	RFS (*n* = 89)	NRFS (*n* = 216)	Z/*χ*^2^ value	*P-*value
New ALI within 28 d, *n* (%)	25 (28.09)	18 (8.33)	20.314	0.000
New AKI within 28 d, *n* (%)	22 (24.72)	13 (6.02)	21.698	0.000
New MV within 28 d, *n* (%)	46 (51.69)	29 (13.43)	49.754	0.000
Length of hospitalization (d)	31 (20, 48.5)	31.5 (20, 47.75)	−0.049	0.961
Morbidity within 28 d, *n* (%)	14 (15.73)	10 (4.63)	10.713	0.001
mRS on discharge (score)	5 (4.5)	4 (2.5)	−3.841	0.000

ALI, acute liver injury; AKI, acute kidney injury; MV, mechanical ventilation.

### 3.3 Comparison of the serum phosphorus level of patients between the RFS and the NRFS group

**Figure 1:**
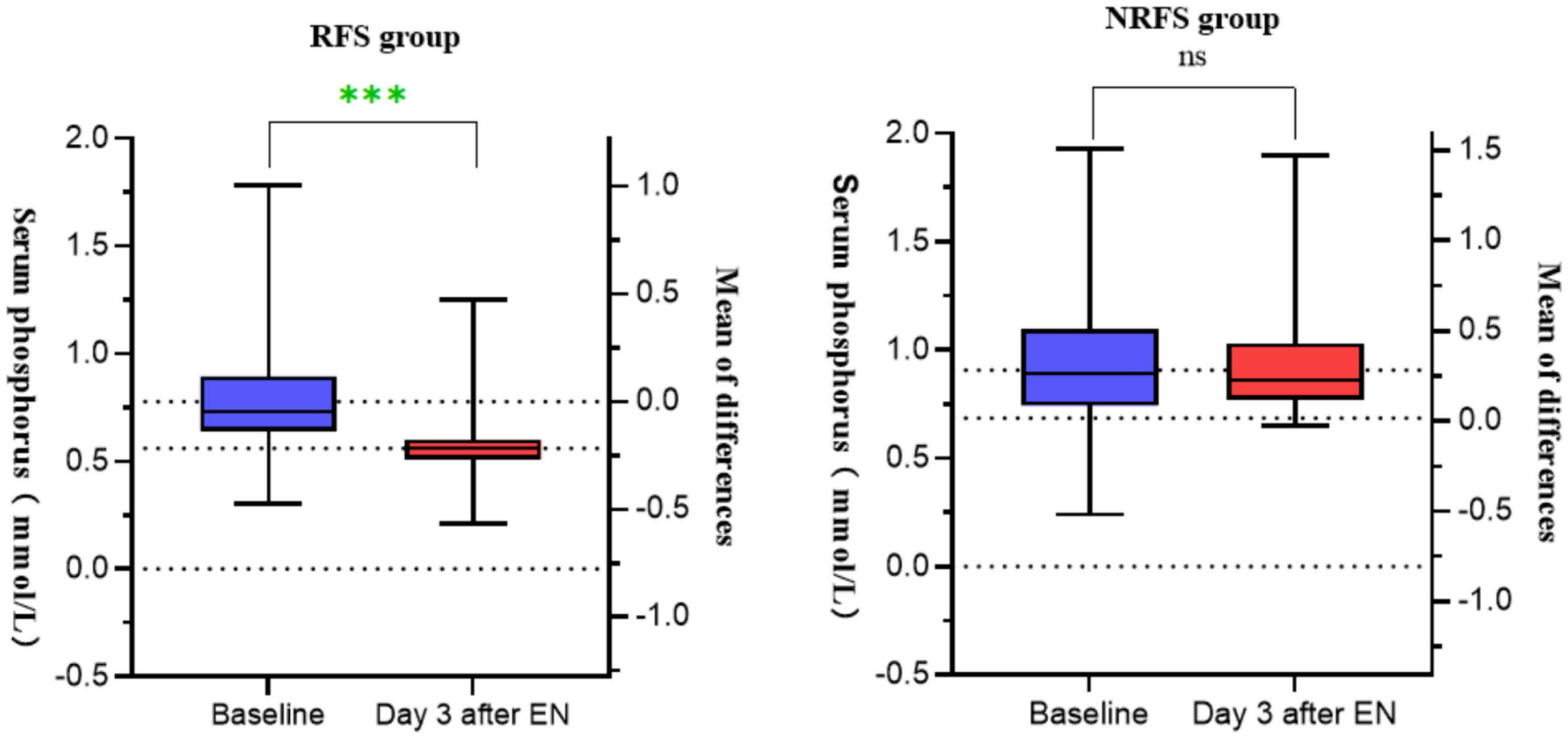
Graph of changes in serum phosphorus of patients before and after EN in the two groups.

The serum phosphorus level in the RFS group decreased significantly after EN (0.78 ± 0.24) compared to before EN (0.56 ± 0.16) (*p* < 0.05), while there was no difference in serum phosphorus levels before and after EN in the NRFS group, which as shown in Figure 1.

### 3.4 Analysis of the correlation between RFS and prognosis of patient with severe stroke

Pearson’s correlation analysis showed that RFS was positively correlated with mRS score (*r* = 0.233, *p* = 0.000), APACHE II score (*r* = 0.307, *p* = 0.000) and SOFA score (*r* = 0.383, *p* = 0.000) on admission in patients with severe stroke.

### 3.5 Risk factors for prognosis of patients with severe stroke

**Figure 2:**
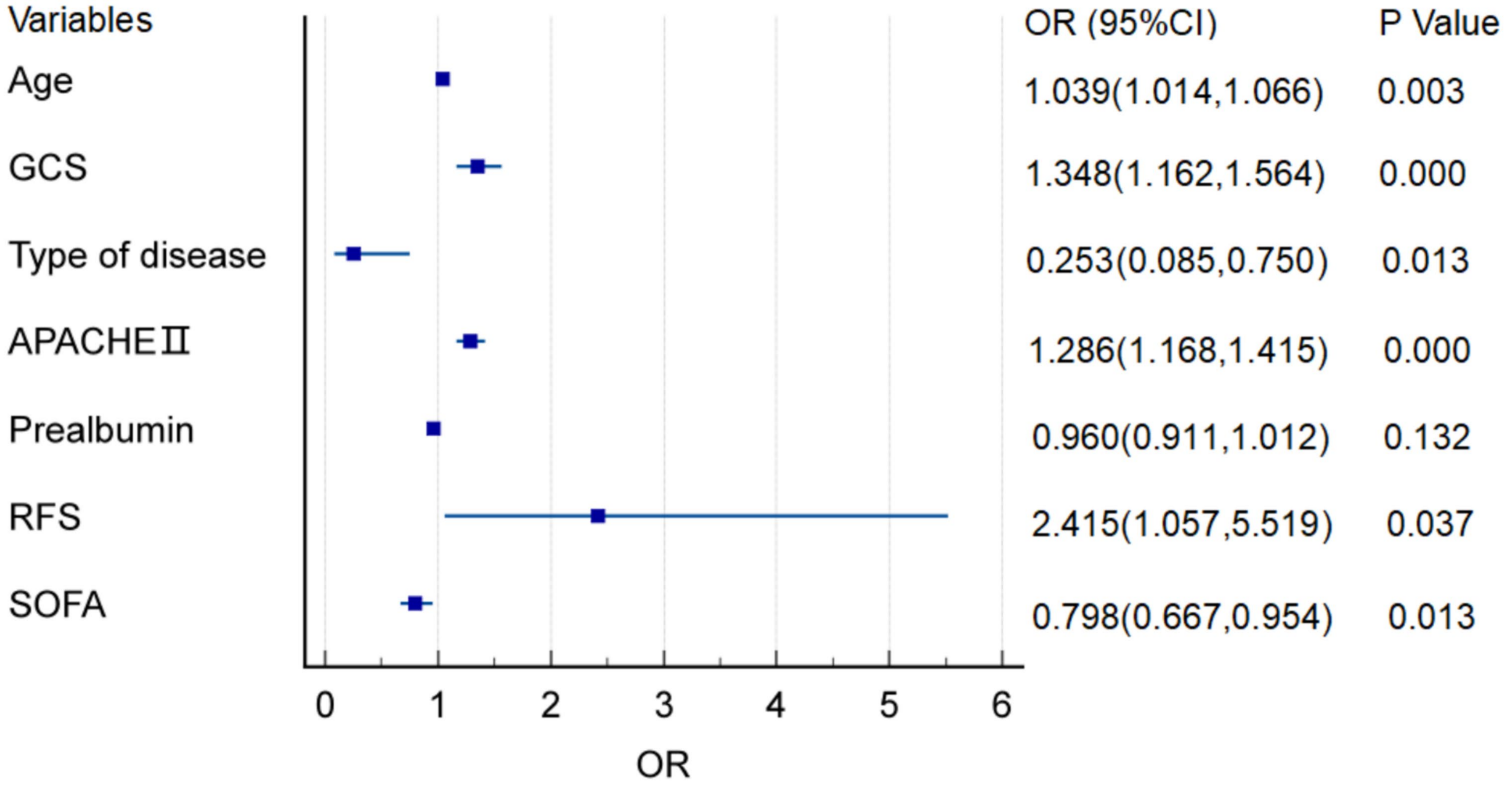
Forest plot of prognostic risk factors in patients with severe stroke.

The prognosis of patients with severe stroke (assigned values: good prognosis = 0, poor prognosis = 1) was the dependent variable, and the independent variables with significance in the univariate analysis (assigned values of haemorrhagic stroke = 0, ischemic stroke = 1, NRFS = 0, RFS = 1, APACHE II score, SOFA score, GCS score, and prealbumin on admission were the measured values) were subjected to the binary logistic regression analysis, and the results are displayed in Figure 2, which showed that age, type of disease, APACHE II score, SOFA score, GCS score and RFS were factors influencing the prognosis of patients with severe stroke (*p* < 0.05).

### 3.6 The value of RFS in predicting poor prognosis in patients with severe stroke

**Figure 3:**
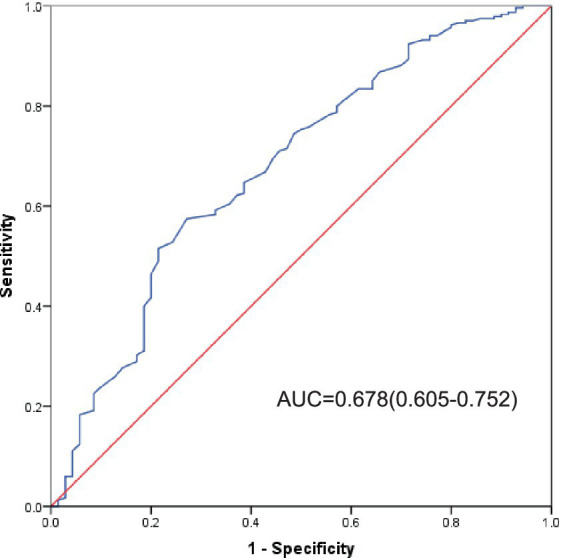
ROC curve of RFS for predicting prognosis in patients with severe stroke.

The AUC of RFS for predicting poor prognosis in patients with severe stroke was 0.678 (95% CI: 0.605–0.752), with a sensitivity of 64.7%, a specificity of 61.4%, and a cut-off value of 0.845, as shown in Figure 3.

### 3.7 Comparison of the survival time for patients between the RFS and the NRFS group

**Figure 4:**
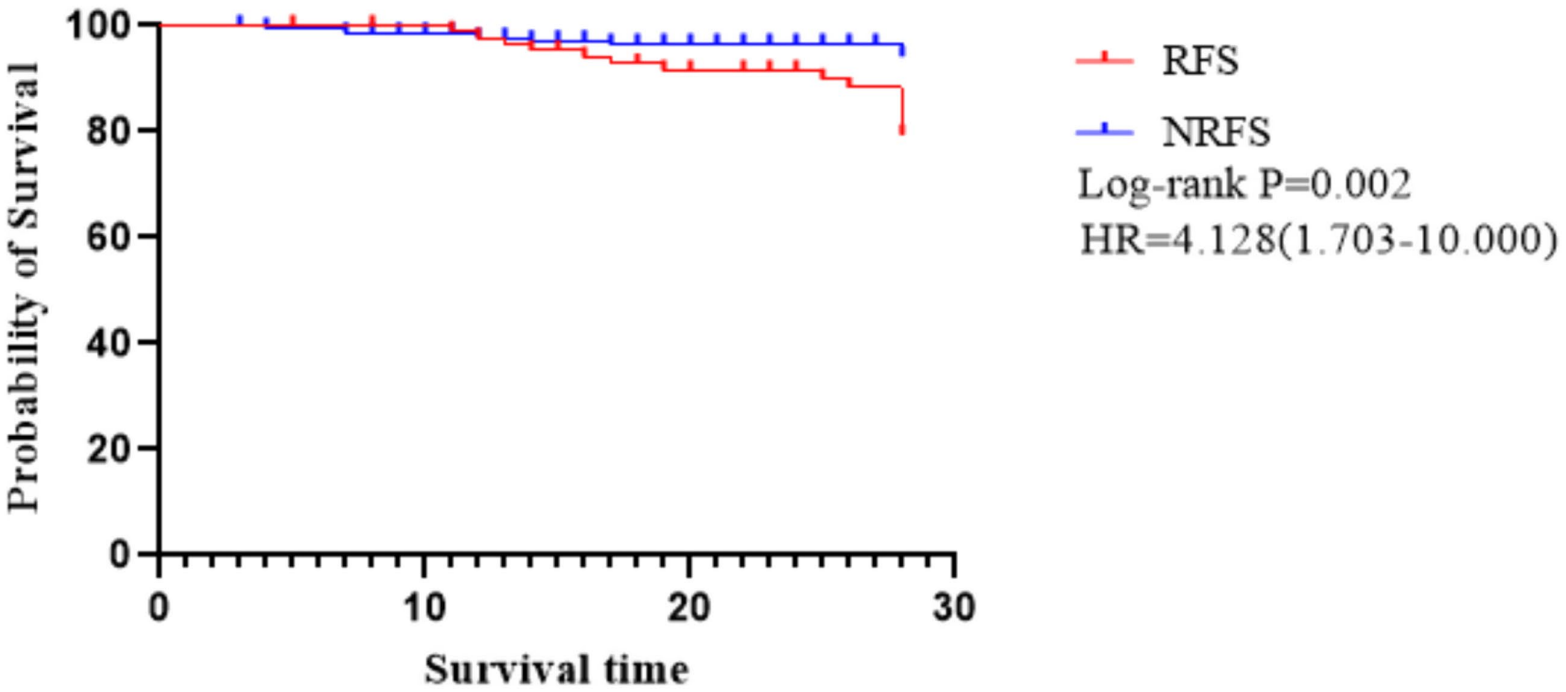
Survival curves for patients with severe stroke.

There was a significant difference in the 28-day mortality between the two groups (Log-rank *p* < 0.001), as shown in Figure 4. Multivariate Cox regression analysis showed a significant correlation between RFS and the mortality of patients (HR = 4.128, 95% CI = 1.703–10.000, *p* = 0.002), and MRS score was an independent risk factor for patient mortality (HR = 1.667, 95% CI = 1.107–2.510, *p* = 0.014).

## 4 Discussion

The results of this study show that the mRS scores are significantly higher in the RFS group than in the NRFS group, that is the incidence of poor prognosis in the RFS group is higher. Hypophosphatemia is the main characteristic of RFS (28), and severe hypophosphatemia is considered a hallmark of RFS occurrence and increases the mortality rate of patients (29). This is consistent with the results of this study, which showed a significant increase in new ALI, AKI, MV and mortality rate within 28 days in patients with concurrent RFS. Phosphates play an indispensable role in the structural integrity of cell membranes, which are essential minerals for several intracellular processes such as glucose metabolism, energy storage, and activation of enzymes and second messengers (30). As malnutrition progresses, the body will continuously consume existing phosphates to continue producing ATP. Phosphate depletion will cause the respiratory muscle dysfunction, and will develop into acute respiratory failure in patients (31), which leads to prolonged mechanical ventilation of the patients (32). In addition, phosphates can regulate the affinity of hemoglobin for oxygen. Hypophosphatemia leads to a decrease in glyceryl diphosphate levels, which increases the affinity of hemoglobin for oxygen, resulting in impaired oxygen release from peripheral tissues and causing tissue hypoxia (33). Besides, hypophosphatemia also leads to a decrease in ATP levels in white blood cells, resulting in the impairment of phagocytosis and bactericidal abilities (34). Therefore, hypophosphatemia causes a decrease in the body’s anti-inflammatory and antioxidant abilities, resulting in acute multi organ dysfunction, and ultimately leading to various adverse clinical outcomes.

However, this study has not yet found that RFS increased the length of hospitalization, which is inconsistent with the findings of Friedli et al. (35), who noted that patients with a diagnosis of RFS had a longer average length of ICU stay. This may be due to the fact that this study included patients with severe stroke and was an analysis of total length of hospitalization. Stroke patients require transition and rehabilitation after transfer out of ICU in addition to symptomatic treatment in the acute phase. While, considering the limitations imposed by the Medicare Board on the length of hospitalization of patients, and physicians usually control the cycle of the patient’s hospitalization, which is the main reason for the lack of difference in the length of hospitalization in this study. In addition, the result of this study shows that the incidence of RFS in severe stroke patients was 29.2%, which is higher than the study by Xiong et al. (14). This may be due to the different definitions of RFS, the different groups of participants, and the different methods of nutritional delivery. Therefore, in the future, there is a need to standardize the definition of RFS so that the incidence of RFS leading to the poor prognosis of patients can be more comparable.

In this study, logistic regression analysis shows that RFS is a risk factor for poor prognosis in patients with severe stroke. This is related to cardiorespiratory dysfunction and metabolic disorders caused by hypophosphatemia (36). When the body is in a long-term state of hunger, it will switch from carbohydrate energy supply to fat and protein energy supply (37). Plasma insulin levels decrease while glucagon increases, and intracellular electrolytes and vitamins such as phosphorus, magnesium, and potassium are consumed in large quantities. Due to concentration and reduced renal excretion, the levels of phosphorus, magnesium, and potassium in the serum may be normal at this time; When fed again, the body changes from a catabolic state to an anabolic state, resulting in an increase in blood sugar, an increase in insulin secretion, and a decrease in glucagon secretion, promoting the synthesis of glycogen, fat, and protein. This process requires the participation of phosphorus, magnesium, potassium, and vitamins. Eventually, electrolytes that were originally almost depleted are transferred from the extracellular space to the intracellular space, further reducing the levels of phosphorus, magnesium, and potassium in the serum, which will lead to functional impairments in respiratory muscle cells, myocardial cells, nerve cells, and various organ cells throughout the body (38). In addition, after nutritional support therapy, the energy supply is converted from fat to carbohydrate, and the process of glucose glycolysis and the formation of adenosine triphosphate has to be phosphorylated, thus leading to the depletion of PO43-. PO43- maintains the structural integrity of the cell membranes and facilitates the segregation of oxygen from haemoglobin, and with the decline in the level of PO43-the supply of oxygen to the blood decreases, and the peripheral tissues are ischemic and hypoxic, which ultimately causes serious complications such as heart failure, arrhythmia, and respiratory muscle dysfunction (39). Therefore, while determining clinical treatment strategies for severe stroke patients, timely targeted measures for RFS may be of great significance in improving the prognosis of severe stroke patients.

The ASPEN guidelines for the prevention and treatment of RFS suggest reducing or stopping feeding for patients diagnosed with RFS after feeding, in order to improve the medium and long-term prognosis of patients (40). However, deliberately reducing the early nutritional intake of critically ill patients can lead to undernutrition, which may increase the incidence of adverse clinical outcomes, such as ventilator failure, stress ulcers, and gut microbiota translocation (41). Therefore, based on the ability to identify clinical prognosis, further improving the diagnostic definition of RFS is of great significance for the early nutritional support of severe stroke patients. Therefore, we believe that based on the predictive ability of RFS for prognosis of patients, the reasonable diagnostic criteria should be further identified. At the same time, combined with the risk factors of RFS proposed by previous studies, it is beneficial to screen high-risk populations as early as possible, so as to choose appropriate nutritional treatment protocol, avoid or reduce the occurrence of RFS, and improve patient prognosis. In this study, the optimal cutoff value of RFS for predicting patient prognosis was 0.845, which is higher than the study by Doig et al. (23), possibly due to differences in the participating population. Doig et al. (23) targeted all critically ill patients, who may have a wider range of diseases, more severe and complex conditions. Therefore, the diagnostic criteria for controlling serum phosphorus levels are more stringent. Besides, the results of this study indicate that the AUC of RFS for predicting the short-term prognosis in patients with severe stroke is limited. This may be due to the severity, complexity, and multiple comorbidities of severe stroke patients, so the fact that RFS induced by nutritional therapy is not the only factor determining patient prognosis. In addition, the diagnostic criteria used in this study are not the gold standard for RFS diagnosis. However, due to the fact that the diagnostic method for RFS in this study mainly relies on changes in serum phosphorus levels, while we strictly followed the inclusion and exclusion criteria when including participants, and excluding those who are prone to interfering with changes in serum phosphorus levels such as renal dysfunction and endocrine disorders. As a result, the participants’ serum phosphorus levels remained relatively stable. Therefore, the results of this study can still demonstrate that RFS demonstrates moderate predictive performance for the short-term clinical prognosis of severe stroke patients.

This study has the following limitations. Firstly, the retrospective nature limits the level of evidence. Relying on existing medical records introduces risks of information bias (e.g., incomplete data) and selection bias. In this study, some variables for which data may not be accurately collected, such as BMI, history of disease, history of medication, etc. In addition, not all patients underwent serum phosphorus testing during the treatment period due to different habits of different doctors. Thus, patients without serum phosphorus test results were excluded. Moreover, the sample selected in this study were from the NISCU, the proportion of patients with ischemic stroke is relatively low, which may lead to some selection bias. Therefore, the results can only indicate association, not prove causality between RFS and prognosis. Secondly, this study is single-center design and convenience sampling method was adopted in selecting the sample, which may be limited by homogeneity of a single region and population, resulting in limitations in sample representativeness. Specifically, differences in nutritional support protocols and patient baseline characteristics across hospitals and regions may limit the generalizability of the findings. Thirdly, this study uses the Doig criteria to define RFS. However, RFS is a syndrome involving multiple electrolyte and metabolic disturbances. A definition centered solely on low serum phosphate may overlook the combined impact of concurrent hypokalemia, hypomagnesemia, and fluid balance disorders, potentially affecting the accuracy of group classification and the assessment of RFS’s true effect. Fourthly, although multivariate analysis was performed, retrospective data may not fully capture and adjust for all significant prognostic confounders, such as the precise severity of the stroke itself (beyond the mRS, e.g., NIHSS at admission), the exact timing and dosage of nutrition initiation, and concomitant infections or multi-organ status. Therefore, this has a certain impact on the accuracy of the results. Finally, this study only analyzed the differences in electrolyte levels between patients after admission and 72 h after EN treatment, but did not analyze subsequent electrolyte changes, which may not fully reflect the trend of electrolyte changes. Besides, this study only analyzed the impact of RFS on the short-term prognosis of patients and did not follow up on the subsequent conditions of patients. Therefore, multicenter, prospective cohort studies are needed to validate long-term outcome (e.g., 90-day survival) in the future. Furthermore, exploring composite diagnostic or risk prediction models that incorporate multiple electrolyte levels, metabolic markers, and clinical features could enhance the accuracy of predicting adverse outcomes.

## 5 Conclusion

RFS is a risk factor for short-term prognosis in patients with severe stroke, which can increase the incidence of poor clinical prognosis. RFS based on the lowest blood phosphorus value holds some predictive value for short-term prognosis of patients. Therefore, refining the severity grading of RFS based on the ability of RFS to predict the clinical prognosis of severe stroke patients is important for the selection of early nutritional intake in patients. In the future, the diagnostic criteria for RFS need to be further clarified and routine phosphate monitoring in high-risk stroke patients during initial enteral nutrition to avoid or reduce the occurrence of RFS during the nutritional treatment process, thereby improving patient prognosis.

## Data Availability

The original contributions presented in the study are included in the article/supplementary material, further inquiries can be directed to the corresponding author.
